# Editorial: Medical neurohumanities: sharing insights from medicine, neuroscience, and music in pediatric care

**DOI:** 10.3389/fnins.2025.1648030

**Published:** 2025-07-22

**Authors:** Artur C. Jaschke, Efthymios Papatzikis, Friederike B. Haslbeck

**Affiliations:** ^1^Department of Psychiatry, Evelyn Perinatal Imaging Centre, Rosie Hospital, University of Cambridge, Cambridge, United Kingdom; ^2^Department of Music Therapy, ArtEZ University of the Arts, Enschede, Netherlands; ^3^Division of Neonatology, University Medical Center Groningen, Groningen, Netherlands; ^4^Early Childhood Education and Care, OsloMet-storbyuniversitetet, Oslo, Norway; ^5^Neonatology, Universitat Zurich, Zürich, Switzerland

**Keywords:** neonatal intensive care unit (NICU), neurohumanities, music therapy, medicine, pediatrics, care

In recent years, the concept of “Medical Neurohumanities” has emerged as a vibrant interdisciplinary field, blending the insights of medicine, neuroscience, and the humanities to deepen our understanding of health, illness, and healing. This Research Topic, “Medical Neurohumanities: Sharing Insights from Medicine, Neuroscience, and Music in Pediatric Care,” brings together diverse perspectives that showcase how integrating knowledge across disciplines can meaningfully enrich pediatric care, particularly for vulnerable populations such as preterm infants, critically ill children and their families.

Neurohumanities have been debated and increasingly applied in medicine, with the vital relationship between music sciences, music therapy, and pediatric care remaining underexplored. On the one hand,

“[…]neurology attempts to understand the biological basis of our humanity. At the same time, neurological conditions profoundly impact our humanity. The practice of neurology requires a curiosity about the person, the disease, and the ability to listen to and hear stories.”

- Carandang (2025; online)

On the other, neurohumanities re-emphasize the humanistic aspects of medical practice and investigate the importance and influence of perspectives, behaviors, and feelings related to the practice of neurology and medical practice at large. Furthermore, they raise self-awareness and the impact of our perspectives on others and explore the intersection of art and neurology medicine and how neurology positively impacts the world.

As our understanding of the young brain expands, creating a shared lexicon that connects music, neuroscience, technological innovations, pediatrics, and healthcare is crucial. By fostering interdisciplinary collaboration, we can deepen our comprehension of the working-mechanisms of music therapy and music medicine for pediatric patients. Sharing insights, approaches, methods and interventions promotes interdisciplinary understanding, enabling the development of more effective, patient- and family-centered healthcare strategies that consider ethical aspects, shared therapeutic goals, care and medical responsibilities. This will lead to a de-disciplined approach, where the expert identifies the boundaries of their discipline, to reach beyond that border toward new frontiers in any given field.

This Research Topic aimed to foster dialogue between scientific and artistic disciplines and highlight innovative approaches where neuroscience, clinical medicine, therapy and music intersect to improve (neuro)developmental as well as clinical outcomes and humanize healthcare experiences. By bridging gaps between empirical evidence and clinical practice, the contributing articles offer new lenses through which pediatric healthcare and development can be viewed, supported, and enhanced.

Several contributions focus on the early developmental period and the unique needs of neonates and their caregivers. For example, Hugoson et al. present the pioneering work on Creative Music Therapy (CMT) in neonatal intensive care units (NICUS), supporting parent-infant bonding and neurodevelopmental trajectories in preterm infants (Haslbeck et al., 2023; van Dokkum et al., 2020; Bos et al., 2021). Their study emphasizes that parental singing during kangaroo care, primarily when facilitated by a trained music therapist, strengthens the parents' sense of coherence by promoting experiences of comprehensibility, manageability, and meaningfulness. Interestingly, this is the first study that interviewed singing parents of an intervention group which received CMT and the control group without CMT support. Parents who received CMT reported a deeper understanding of how singing facilitates attachment and enhances self-esteem, reflecting improvements in perceived manageability and comprehensibility. While parents in the control group also expressed joy in singing and noted positive effects on family wellbeing, these experiences were less pronounced, not described as a healing experience and catharsis as in the CMT group and started far later during the NICU trajectory since many parents were not able to raise their voice for singing during the first weeks of their NICU stay. However, particularly during these first weeks, parental anxiety, stress and trauma may evolve and imprint so that therapeutic support, such as CMT, may act as a preventive health intervention for these families as indicated in Kehl et al. (2021). Complementing this, Dewan et al. examined the impact of live music therapy on the parental stress levels of preterm infants during the neonatal care period. Their findings highlight how music therapy can reduce subjectively perceived parental stress and more objective indicators of stress, such as parental cortisol levels.

While the improvements on parent child bonding are compelling, there are additional benefits from music interventions on emotional and neurodevelopmental outcomes in preterm infants. Filippa et al., has tracked infants at 12 and 24 months post intervention revealing enhancements in emotional regulation and neurodevelopmental milestones, underscoring the importance pf early auditory stimulation in at risk-populations.

The Euterpe music therapy methodology has been introduced as a structured approach to integrating music therapy in pediatric care (Liuzzi and D'Arienzo et al.). This methodology includes detailed procedural algorithms designed to standardize and optimize music therapy interventions, ensuring consistency and efficacy in clinical settings.

From a broader theoretical perspective, Stige (2012) offers a conceptual framework for “musicking” in healthcare contexts, linking neurobiological mechanisms with psychosocial processes. He advocates for a relational understanding of music as a co-created, embodied activity that transcends traditional therapy models, opening possibilities for its application in pediatric and family-centered care.

These articles illuminate the transformative potential of the Medical Neurohumanities in pediatric care. They illustrate how integrating arts-based practices, relational neuroscience, and humanistic inquiry can not only optimize developmental outcomes but also rehumanise healthcare environments that are often dominated by technology and protocols.

Innovative research exploring the feasibility of using clinical EEG to recognize music in children, demonstrates the potential for non-invasive monitoring of auditory processing. This approach as is described below, could pave the way for personalized music therapy strategies tailored to individual neural processes.

Three critical tensions persist despite the demonstrable clinical promise illustrated across this Research Topic. First, methodological heterogeneity—ranging from the single-center, low-powered EEG feasibility study by Bower et al. to the highly “protocolized” yet context-bound Euterpe algorithms detailed by Liuzzi and D'Arienzo et al. and Liuzzi, Bompard et al.—impedes meta-analytic synthesis and obscures dose–response relationships. Second, outcome measures remain fragmented: while Dewan et al. document acute biobehavioural stress attenuation in parents, van Dokkum et al. reveal downstream respiratory and mood sequelae linked to NICU stress that current interventions leave mostly unaddressed. Third, scalability is constrained by limited implementation science; the carefully staged guidelines for early parental vocal contact advanced by Filippa and Kuhn, although pragmatic, have yet to be benchmarked against health-economic or cross-cultural metrics.

Consequently, future research within Medical Neurohumanities must transition from proof-of-concept to precision, leveraging digital phenotyping (Insel, 2017), multimodal neuroimaging (Papatzikis, 2024), and federated data repositories with advanced Machine Learning algorithms to both establish reproducible biomarkers of musical efficacy and inform novel interventions and control the clinical environment on the ward and at bedside (Jaschke, 2025; Kunikullaya et al., 2025; Jaschke et al., 2024; Jaschke and Bos, 2023). Multi-site adaptive trials—anchored in standardized clinically relevant core outcome sets and harmonized intervention taxonomies—could clarify the timing and sensitivity periods across the developmental trajectory, as suggested by the 12- and 24-month emotional modulation evidenced by Filippa et al.. In parallel, implementation laboratories should interrogate contextual moderators, integrating caregiver-reported or family-centered feasibility and family health indicators with real-time physiological analytics to inform just-in-time adaptive interventions. Finally, the field would benefit from consensus on an ontological lexicon that unites neuroscientific constructs (e.g., predictive coding, interoceptive attunement) with humanities-derived notions of meaning-making, thereby fulfilling the transdisciplinary mandate of this Special Research Topic and accelerating the translation of embodied musical experiences into equitable, sustainable pediatric care.

This Research Topic invites healthcare professionals, researchers, and artists alike to envision new paradigms of pediatric care—ones that are attuned to the biological, emotional, and relational complexities of early human life. As the field of Medical Neurohumanities continues to evolve, it holds promise for fostering more holistic, compassionate, and effective models of care for children and their families.

This Research Topic underscores the growing recognition of the intersection between the arts and sciences in pediatric care. By highlighting the therapeutic benefits of music and auditory interventions, it advocates for a more holistic approach to pediatric healthcare—one that considers children's emotional and developmental needs alongside traditional medical treatments. Integrating music therapy into clinical practice enriches the healthcare experience for parents and families and opens avenues for future research and innovation in pediatric and neonatal care.

We hope this Research Topic catalyzes further interdisciplinary collaborations and inspires innovative practices that honor both the science and the art of healing.
